# The Correlations between Health-Related Quality of Life Changes and Pain and Anxiety in Orthodontic Patients in the Initial Stage of Treatment

**DOI:** 10.1155/2015/725913

**Published:** 2015-01-22

**Authors:** Jing Wang, Xiaoshan Tang, Yun Shen, Guangwei Shang, Li Fang, Raorao Wang, Yuanzhi Xu

**Affiliations:** Department of Stomatology, Shanghai Tenth People's Hospital, Tongji University School of Medicine, No. 301, Middle Yanchang Road, Shanghai 200072, China

## Abstract

This study aimed to assess generic health-related quality of life (HRQoL), pain intensity, and anxiety levels and the relationship between the three aspects in healthy young Chinese orthodontic patients in the early stage of orthodontic treatment. We enrolled 252 eligible participants (10–29 years old) to complete validated Chinese versions of questionnaires, including the State-Trait Anxiety Inventory (S-AI), the visual analogue scale (VAS), and the Short-Form 36-Item Health Survey (SF-36) at baseline and on days 1, 2, 3, 7, 14, and 30 after initial archwire placement (SF-36 only at baseline and day 30). The response rate was 96% (243 of 252). SF-36 had moderate reliability (Cronbach's alpha coefficient exceeding 0.7, good fit on day 30). Statistical significant changes were observed in physical function (*P* < 0.01), body pain (*P* = 0.01), and general health (*P* < 0.01) domains. Spearman correlation coefficients for SF-36 with S-AI were −0.131~−0.515 (*P* < 0.05); SF-36 with VAS were −0.141~−0.273 (*P* < 0.05), indicating significant but moderate negative correlations between HRQoL and pain/anxiety. Overall, the application of SF-36 in assessing HRQoL is reluctantly suitable for young Chinese orthodontic patients in the early stage of orthodontic treatment. Early treatment-related pain and anxiety are important factors in HRQoL.

## 1. Introduction

It is acknowledged that health contributes to quality of life (QoL), known as health-related quality of life (HRQoL), which is now recognized as a significant parameter for patient assessment in nearly every field of physical and mental healthcare, including orthodontics [1, 2]. There have been a growing interest and need to evaluate orthodontic treatment in terms of QoL with standardized measurement tools, because it has far-reaching implications for both clinical practice and research, by providing “informed consent” and accurate assessment of overall health benefit with consideration of treatment morbidity [3]. Moreover, it helps clinicians to identify what suffering the patients must experience in the treatment and to integrate these factors when recommending treatment options [4].

Previous studies have found that the severity of malocclusion and esthetic impairment was higher in adolescents with orthodontic treatment, resulting in a worse QoL score compared with the age-matched peers who were not seeking orthodontic treatment [5]. Besides, the motivation and attitude of treatment seeking and the outcomes from orthodontic care are related to QoL issues [6]. Notably, the treatment process* per se* also has significant influence on QoL, because of some treatment-related side effects of orthodontic treatment [2, 7, 8] especially in the fixed orthodontic appliance therapy [9, 10]. Early studies reported that many patients, especially adolescents, have difficulty adjusting to the treatment due to pain, anxiety, and distress [8] and feel somewhat embarrassed about the esthetic change in appearance with fixed orthodontic appliances [9]. But with the popularity of orthodontic treatment in recent years, the aesthetic factor's influence on life quality becomes smaller since this temporary change in appearance is gradually accepted by patients and their peers [11]. Nowadays, the physical symptoms such as pain and discomfort still remain the chief concern for both patients and clinicians. Moreover, the process of pain is closely associated with anxiety which is another frequently reported side effect in orthodontic treatment [12–14].

Orthodontic pain is most intensely felt in 24 to 48 h after force application and dies away in a week [7]. This may cause serious deterioration in HRQoL. According to our clinical experience and previous finding [15], the initial stage in the orthodontic treatment is quite important to the whole process since it is a critical time for the patients to adjust themselves to the treatment. A majority of withdrawal patients attribute their discontinuation to the unbearable pain during this time [7]. Some researchers reported that the period of greatest change in HRQoL occurs during the first month of orthodontic treatment by continuously assessing HRQoL for at least 6 months [10, 16, 17]. Based on these results, we set our observation point in the initial month of orthodontic treatment to learn the changes in patients' life quality in the present study.

The Medical Outcomes Study (MOS) Short-Form 36-Item Health Survey (SF-36) is one of the most frequently used generic health measure instruments for the assessment of HRQoL [18]. It covers the 8 most commonly measured health concepts in the form of 8 scales of physical, mental, and social functions. Factor analytic studies and clinical trials have confirmed the validity and reliability of the 8 scales among diverse populations of different races or cultural backgrounds in different languages [19–23]. In addition, SF-36 has been reported to be effective in various clinical scenarios as a generic measure of health status with a potential for widespread applications in chronic conditions [18].

Recent years have witnessed the increasing use of the SF-36 in orthognathic and orthodontic-surgical treatment to determine changes in HRQoL [3, 24]. The data from our previous study demonstrated a significant alleviation in the body pain domain in the SF-36 [15]. Therefore, we suppose that SF-36 may be suitable in assessing generic HRQoL in orthodontic practice.

To assess generic HRQOL and to learn the contribution from pain intensity and anxiety level to the HRQoL changes in healthy young Chinese orthodontic patients after initial archwire placement, we measured the levels of pain, anxiety, and quality of life at the baseline and during the treatment. Relevant correlation analyses were also performed.

## 2. Material and Methods

### 2.1. Subjects

We conducted the study between December, 2012, and July, 2013, at the Department of Stomatology, Shanghai Tenth People's Hospital, Tongji University School of Medicine. Patients seeking orthodontic treatment at the department were screened for suitability. All adult subjects and the parents of minor subjects were given detailed information about the study and signed written consent. The protocol was approved by the Ethics Committee of Shanghai Tenth People's Hospital (number 2012-Res-036).

The inclusion criteria were subjects with a need for orthodontic treatment (self-perceived) and about to undergo fixed orthodontic appliance therapy (straight-wire technique). Patients were excluded if they had severe malocclusion; previous orthodontic treatment; recent toothache; periodontal diseases (periodontal pocket > 3 mm); untreated dental caries (cavitated lesions); infectious diseases and/or systemic diseases; or overanxiety, as confirmed by the Trait-Anxiety Inventory (T-AI) score (male ≥56; female ≥57) and the State-Anxiety Inventory (S-AI) score [25] (male ≥53; female ≥55).

### 2.2. Data Collection and Instruments

The routine information collected included age, sex, city of origin, and clinical diagnosis. All subjects were diagnosed with mild to moderate malocclusion based on normative need indices and underwent careful oral examinations before the interventions. S-AI, VAS, and SF-36 were administered to the subjects at baseline and on days 1, 2, 3, 7, 14, and 30 after initial archwire placement (SF-36 only at baseline and day 30).

### 2.3. Quality of Life

Subjects were given self-administered Chinese version of the SF-36 [22], which consists of 36 items measuring physical and mental status under 8 health domains: (1) physical functioning (PF); (2) role limitations due to physical problems (RP); (3) body pain (BP); (4) social functioning (SF); (5) general mental health (MH); (6) role limitations because of emotional problems (RE); (7) vitality (VT); and (8) general health perceptions (GH). Scores for the 8 domains range from 0 to 100, with higher scores indicating better HRQoL. A scale score would be declared absent when more than half of its items were missing. If less than half of the items were missing, the mean score of the nonmissing items would be regarded as representative of the scale.

### 2.4. Anxiety Level and Pain Intensity

The ST-AI is a 40-item Likert scale which assesses separate dimensions of “state” anxiety (items l–20) as well as “trait” anxiety (items 21–40) [25]. Pain intensity was measured by a 100 mm visual analogue scale (VAS) ranging from 0 = no pain at all to 10 = worst pain possible [26].

After initial archwire placement, VAS and S-AI scores were collected on days 1, 2, 3, 7, 14, and 30. The SF-36 scores were collected on days 30 after initial archwire placement.

### 2.5. Evaluation of Sample Size

Data from our previous study [15] showed that BP domain in the control group presented M (baseline) = 79.97, SD (baseline) = 12.63; M (1 m) = 74.21, SD (1 m) = 15.51. Therefore, we set matched-pair design formula for sample size evaluation:

(1) $$\begin{array}{c} n={\left[\frac{\left(u_{\alpha }+u_{\beta }\right)}{\delta /\sigma }\right]}^{2}+\frac{1}{2}u_{\alpha }^{2}. \end{array}$$

### 2.6. Statistical Analysis

The data were entered into a database by two independent data managers, and any inconsistencies were corrected by EpiData verification. Overall summary and domain scores for SF-36 and S-AI, measured at different time intervals, were derived according to their scoring algorithms [18–20, 23]. Changes in the 8-dimension scores of SF-36 at baseline and day 30 were evaluated by the Wilcoxon signed-rank test.

The correlations between the outcomes of SF-36, with VAS and S-AI, were tested by Spearman rank correlation. SPSS version 16.0 (SPSS, Chicago, IL, USA) was used to perform the analysis.

## 3. Results

### 3.1. Characteristics of Participants

We enrolled 252 eligible participants requiring fixed orthodontic treatment. Of these, 243 completed the initial treatment and assessments at all time-points (response rate: 96%). Patients' clinical and sociological characteristics are summarized in Table 1. Among the subjects, the majority were female, (58.85%). One hundred and forty subjects were adolescents (10–16 years, 60.91%) and 95 were young adults (17–29 years, 39.09%). No significant differences were observed in the baseline HRQoL assessment of those who completed all assessments (included in the analyses) and those who failed to complete all assessments (excluded from the analyses) (*P* < 0.05).

### 3.2. Descriptive Data of SF-36

Statistically significant changes in SF-scores were observed in 3 domains before and after initial archwire placement: PF (*P* < 0.01), BP (*P* = 0.01), and GH (*P* < 0.01) as displayed in Table 2. We were interested in the changed aspects in HRQoL; therefore, the three domains were focused on in later analyses.

### 3.3. Reliability and Validity of SF-36

The internal reliability Cronbach's alpha coefficient for the SF-36 at baseline was 0.734 and on day 30 was 0.781. The theoretical model had good fit on day 30 of the orthodontic treatment demonstrated by results of confirmatory factor analysis with the main evaluation indexes, NFI (normed fit index) = 0.907, CFI (comparative fit index) = 0.945, and RMSEA (root mean square error of approximation) = 0.072, while it had acceptable fit for data at baseline, NFI = 0.834, CFI = 0.874, and RMSEA = 0.094. This indicates that the SF-36 was a reliable and valid instrument to measure the HRQoL in the early stage of orthodontic treatment.

### 3.4. Pain Intensity and Anxiety Levels

**Figure 1:**
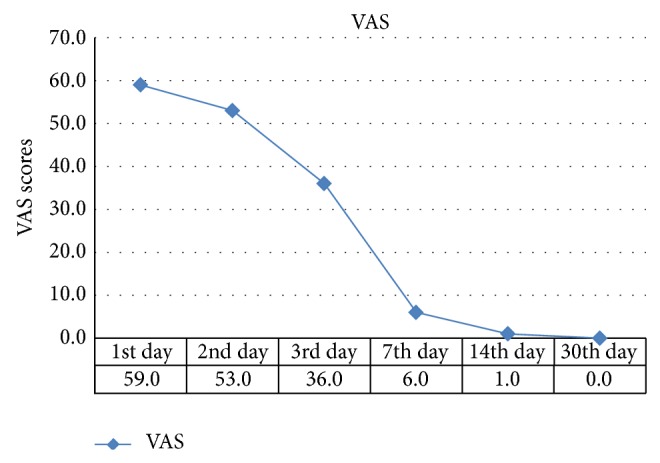
Median of VAS scores in the study subject.

**Figure 2:**
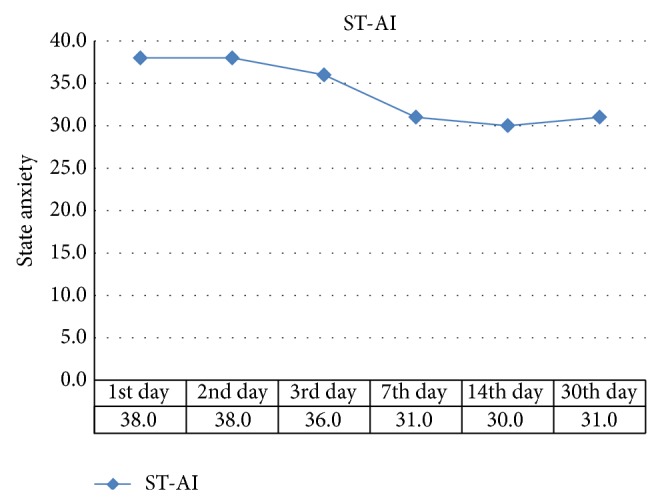
Median of state anxiety scores in the study subject.

Changes in pain scores and anxiety scores on each time-point are shown in Figures 1 and 2, respectively. A significant decrease can be observed in the VAS scores, while the reduction in S-AI scores was relatively slight. This indicated that patients felt pain gradually dying out while remaining a relatively high level of anxiety.  Table 3 presents the correlations between VAS and S-AI scores at 6 time-points. Except for the scores on day 30, statistically significant correlations were observed, and the *r* maximum was 0.333 (*P* < 0.001) on the 14th day, indicating that the VAS score was positively related to the state anxiety score, but the correlation was a weak one (Table 3).

### 3.5. Correlation between HRQoL and Pain Intensity/Anxiety Levels

To investigate the influence of pain intensity on patients' life quality, we compared the initial pain intensity which was most intensely felt (Figure 1) with the dominant changes in HRQoL by assessing the relationship of VAS scores in the first week with the scores from the PF, BP, and GH domains in the SF-36 on day 30. As for the relationship between HRQoL and anxiety levels, we compared the SF-36 scores and S-AI scores.

Results are shown in Table 4. Spearman correlation coefficients of PF, BP, and GH scores and VAS scores ranged from −0.141 to −0.273 (*P* < 0.05), indicating that the VAS score was negatively related to the SF-36. The correlation coefficients of HRQoL scores and S-AI scores showed relatively higher values ranging from −0.131 to −0.515 (*P* < 0.05).

## 4. Discussion

This cross-sectional study introducedthe SF-36 for the first time in China as a method of assessing the health-related quality of life in patients with initial orthodontic treatment. The results showed significant changes in SF-36 scores before and after initial archwire placement in PF, BP, and GH domains (Table 2), indicating that patients' lives had indeed been affected by orthodontic treatment, which is consistent with earlier findings that wearing fixed orthodontic appliances has an impact on HRQoL [4, 15, 16]. This finding helps to inform patients of the likely consequential influence in orthodontic treatment on their lives and thus realistic expectations of treatment may be accepted by patients and parents.

The response rate to the study was high (96%), indicating the feasibility of utilizing multiple patient-oriented psychology and cognition measures in the clinical setting. Incorporating patient perceptions or mental state, reflected by VAS, S-AI, and so forth, is significant in treatment planning, decision-making, and evaluation of treatment outcomes [9, 21]. Although such assessments are time-consuming, they can provide a global view of patients' experiences which should not be neglected in the treatment decision-making process and in assessing the outcome of treatment according to what patients experience during treatment [2, 15, 16].

It has been noted that generic measures may contribute to the optimized orthodontic treatment for improving HRQoL. Generic instruments usually address areas concerning general well-being without focusing as much on specific diseases* per se*. The main advantage of using generic measures is that they facilitate the comparison of various conditions affecting HRQoL [18]. SF-36 was used as a generic measure in the present study because it is acknowledged to be the most widely evaluated generic health outcome measure and has been well-validated in dental studies [27].

We found a significant correlation between the changes of SF-36 (especially in GH and BP domains) and the variation of VAS in the initial stage of orthodontic treatment (Table 4). Orthodontic pain is felt most severely in the first 24 hours after treatment and decreases on the third day [7]. Variations in SF-36 and VAS scores reflected the changes in HRQoL during the course of treatment. This concurs with the results from earlier studies [8, 15, 16]. Our observations spanned 30 days because a normal visit interval in orthodontic treatment is about 4 to 6 weeks, and this observation point was designed to avoid the influence of treatment appliances, force differences, and baseline data assimilation for different orthodontic procedures [15].

We also found significant consistency between SF-36 and S-AI, indicating that the deterioration in global health is related to anxiety state. The correlation analysis showed a higher *r* value between HRQoL with anxiety than that with initial pain intensity (Table 4). This could be explained by the development of orthodontic pain and state anxiety as shown in Figures 1 and 2. Pain intensity dropped more fiercely than anxiety while the latter one remained at a relatively high level. Moreover, the positive correlations between pain intensity and anxiety levels suggested that anxiety is probably induced by ongoing orthodontic pain (Table 3). This concurs with other studies that reported that pain perception can impact patients' psychological well-being due to the persistent discomfort and the fact that most orthodontic patients are adolescents who are already experiencing significant psychological transformation and are sensitive to pain [28]. On the other hand, Sari et al. reported that the state anxiety levels of patients who are about to start orthodontic treatment are high [14], and it may be due to the high expectation of patients or parents which is likely to influence anxiety levels and thus affect self-reported HRQoL [5]. If the SF-36 can be integrated as a monitoring index in the process of orthodontic treatment, it will be timely to find and solve these problems in HRQoL. Orthodontists are also suggested to provide patients with psychological auxiliary to improve the anxiety levels.

The assessments of HRQoL hugely depend on a subject's own experiences and perceptions and it is unclear whether these statically significant changes observed in many cases are clinically significant. As more research work is undertaken in orthodontics with standardized HRQoL assessment measures, our understanding of the relative concept and how to interpret such data will be improved [4, 16].

Compared with previous related studies [19, 21–23], our study sample was relatively small and limited in terms of heterogeneity of age or geographical distribution (most participants were young people from the Shanghai district). This may compromise the credibility of applying SF-36 in orthodontics. Because the majority of orthodontic patients are young people and our study was time- and region-restricted, we were unable to gain convincing results for reliability and validity analysis in this study subject group. Therefore, further multicentered research involving a larger and more heterogeneous study sample is needed.

## 5. Conclusions

This study highlights significant changes in HRQoL following initial orthodontic treatment. Administration of generic questionnaires of HRQoL reminds us to have a thorough evaluation on the impact of orthodontic treatment on patients. Pain intensity and anxiety have certain relationship with HRQoL.

## Figures and Tables

**Table 1:** Means and standard deviations of the scores of the SF-36 domains after the initial archwire placement in the study participants.

	Physical functioning		Physical role		Body pain		General health		Vitality		Social functioning		Emotional role		Mental health	
	mean	SD	mean	SD	mean	SD	mean	SD	mean	SD	mean	SD	mean	SD	mean	SD
All	95.62	10.45	88.31	25.44	77.09	14.49	80.25	16.82	74.83	15.24	90.60	12.89	75.98	69.63	79.21	15.37
10–16 years	94.90	11.76	90.78	22.46	78.20	14.32	84.08	16.06	77.60	15.44	92.40	10.91	84.10	82.53	82.17	14.51
17–29 years	96.74	7.91	84.47	29.21	75.37	14.67	74.28	16.32	70.51	13.93	87.80	15.12	63.33	39.56	74.60	15.60
Male	94.95	11.97	87.10	26.49	74.95	14.61	80.81	15.30	73.53	14.75	89.71	13.08	72.49	34.71	78.11	15.93
10–16 years	94.08	12.54	87.25	25.45	74.22	14.43	83.28	14.52	75.67	14.66	90.90	12.02	74.82	32.95	80.53	15.23
17–29 years	96.25	11.08	86.88	28.30	76.05	14.99	77.10	15.88	70.33	14.50	87.93	14.50	69.00	37.35	74.48	16.45
Female	96.08	9.25	89.16	24.75	78.59	14.27	79.86	17.85	75.73	15.55	91.22	12.76	78.42	86.07	79.98	14.97
10–16 years	95.45	11.24	93.18	19.96	80.92	13.67	84.63	17.09	78.92	15.89	93.42	10.03	90.43	103.30	83.28	13.98
17–29 years	97.09	4.48	82.73	30.00	74.87	14.55	72.24	16.47	70.64	13.64	87.71	15.68	59.20	40.93	74.69	15.11

**Table 2:** Comparison of scores of SF-36 domains at baseline and day 30 after initial treatment^*^.

	SF-36 (baseline), SF-36 (30th day)		SF-36 (baseline), SF-36 (30th day)		*Z* value	*P* value
	Median	Interquartile range	Mean	SD		
PF	0	5	−1.276	9.339	−3.48	<0.01^**^
RP	0	0	−0.045	30.742	−0.26	0.8
BP	0	16	2.695	15.927	−2.71	0.01^**^
GH	−2	15	−4.276	15.583	−4.67	<0.01^**^
VT	0	20	−0.403	15.154	−0.26	0.8
SF	0	24	0.333	15.160	−0.24	0.81
RE	0	33	−3.021	91.667	−1.29	0.2
MH	0	20	−0.955	16.418	−1.48	0.14

^*^Analysis of the 8 domains scores of SF-36 before and after the initial treatment by Wilcoxon signed-rank test.

^**^The differences of domain scores before and after the initial treatment were statistically significant (*P* < 0.05).

(1) Physical functioning: PF; (2) role limitations due to physical problems: RP; (3) body pain: BP; (4) social functioning: SF; (5) general mental health: MH; (6) role limitations because of emotional problems: RE; (7) vitality: VT; and (8) general health perceptions: GH.

**Table 3:** Means (SD) and correlation analysis results of VAS scores and state anxiety scores in the study subject^*^.

	1st day	2nd day	3rd day	7th day	14th day	30th day
VAS	59 (28)	53 (31)	36 (35)	6 (16)	1 (5)	0 (2)
State anxiety	38 (15)	38 (17)	36 (15)	31 (13)	30 (15)	31 (14)
*r*	0.171	0.246	0.189	0.310	0.333	0.125
*P* value	0.008^**^	<0.001^**^	0.003^**^	<0.001^**^	<0.001^**^	0.055

^*^Analysis of the relevance between VAS scores and state anxiety scores on each observation point by Spearman rank correlation.

^**^The rank correlation coefficients were statistically significant (*P* < 0.05).

**Table 4:** Correlation analysis of SF-36 domain scores between VAS and S-AI scores in the study subjects^*^.

	PF-VAS		BP-VAS		GH-VAS		PF-SAI		BP-SAI		GH-SAI	
	*r*_*s*_	*P*	*r*_*s*_	*P*	*r*_*s*_	*P*	*r*_*s*_	*P*	*r*_*s*_	*P*	*r*_*s*_	*P*
1st day	−0.141	0.029^**^	−0.033	0.613	−0.109	0.092	−0.109	0.090	−0.243	<0.001^**^	−0.354	<0.001^**^
2nd day	−0.124	0.055	−0.133	0.040^**^	−0.161	0.013^**^	−0.204	0.001^**^	−0.230	<0.001^**^	−0.369	<0.001^**^
3rd day	−0.115	0.076	−0.153	0.018^**^	−0.170	0.008^**^	−0.207	0.001^**^	−0.143	0.026^**^	−0.360	<0.001^**^
7th day	−0.048	0.462	−0.139	0.032^**^	−0.212	0.001^**^	−0.166	0.010^**^	−0.268	<0.001^**^	−0.413	<0.001^**^
14th day	−0.045	0.487	−0.151	0.020^**^	−0.273	<0.001^**^	−0.236	<0.001^**^	−0.194	0.002^**^	−0.515	<0.001^**^
30th day	−0.012	0.857	−0.143	0.028^**^	−0.222	0.001^**^	−0.205	0.001^**^	−0.131	0.042^**^	−0.466	<0.001^**^

^*^Analysis of the relevance between SF-36 domain scores (on day 30) and VAS/state anxiety scores on each observation point by Spearman rank correlation.

^**^The rank correlation coefficients were statistically significant (*P* < 0.05).
